# MAP4K Family Kinases and DUSP Family Phosphatases in T-Cell Signaling and Systemic Lupus Erythematosus

**DOI:** 10.3390/cells8111433

**Published:** 2019-11-13

**Authors:** Huai-Chia Chuang, Tse-Hua Tan

**Affiliations:** Immunology Research Center, National Health Research Institutes, Zhunan 35053, Taiwan; cinth@nhri.edu.tw

**Keywords:** SLE, DUSP, MAP4K, MAPK, MKP, T cells

## Abstract

T cells play a critical role in the pathogenesis of systemic lupus erythematosus (SLE), which is a severe autoimmune disease. In the past 60 years, only one new therapeutic agent with limited efficacy has been approved for SLE treatment; therefore, the development of early diagnostic biomarkers and therapeutic targets for SLE is desirable. Mitogen-activated protein kinase kinase kinase kinases (MAP4Ks) and dual-specificity phosphatases (DUSPs) are regulators of MAP kinases. Several MAP4Ks and DUSPs are involved in T-cell signaling and autoimmune responses. HPK1 (MAP4K1), DUSP22 (JKAP), and DUSP14 are negative regulators of T-cell activation. Consistently, HPK1 and DUSP22 are downregulated in the T cells of human SLE patients. In contrast, MAP4K3 (GLK) is a positive regulator of T-cell signaling and T-cell-mediated immune responses. MAP4K3 overexpression-induced RORγt–AhR complex specifically controls interleukin 17A (IL-17A) production in T cells, leading to autoimmune responses. Consistently, MAP4K3 and the RORγt–AhR complex are overexpressed in the T cells of human SLE patients, as are DUSP4 and DUSP23. In addition, DUSPs are also involved in either human autoimmune diseases (DUSP2, DUSP7, DUSP10, and DUSP12) or T-cell activation (DUSP1, DUSP5, and DUSP14). In this review, we summarize the MAP4Ks and DUSPs that are potential biomarkers and/or therapeutic targets for SLE.

## 1. Introduction

Both genetic and environmental factors contribute to the clinical heterogeneity of autoimmune diseases [1,2]. Innate immune responses cooperate with adaptive immune responses to induce autoimmune responses; therefore, multiple immune cells—including dendritic cells, neutrophils, macrophages, innate lymphoid cells, T helper cells, cytotoxic T cells, B cells, and Treg cells—are involved in the pathogenesis of autoimmune diseases [1]. Depending on the involvement of damaged tissues, autoimmune diseases are classified as either organ-specific diseases (e.g., multiple sclerosis, type I diabetes, and inflammatory bowel disease) or systemic diseases (e.g., systemic lupus erythematosus, rheumatoid arthritis, and Sjögren’s syndrome) [1].

Systemic lupus erythematosus (SLE) is a severe and even fatal autoimmune disease; SLE patients display pathogenic autoantibody production and multiple organ failures [3]. Inflammatory cytokines play an important role in the pathogenesis of autoimmune diseases. In particular, interleukin 17A (IL-17A) plays a critical role in SLE pathogenesis [4,5,6,7,8,9,10,11]. Several biologic agents have been used to treat autoimmune diseases [12,13,14,15,16,17]; however, the development of an effective therapeutic approach for SLE is very challenging due to the complexity and heterogeneity of the disease [4]. Over the past 60 years, only one therapeutic drug, belimumab/anti-BAFF antibody, has been approved for SLE treatment by the U.S. Food and Drug Administration (FDA) [13]. Even so, belimumab is useful only for SLE patients with modest symptoms, and its effect diminishes over the course of 72 weeks [18]. Thus, novel drug targets for effective treatment of SLE are needed [18]. Besides B cells, T cells also play pivotal roles in the pathogenesis of SLE [19]. Dysregulation of T-cell-mediated immune responses leads to enhanced production of pro-inflammation cytokines and autoantibodies, as well as chemokine-induced macrophage/neutrophil overactivation. Therefore, a better understanding of the T-cell-mediated SLE pathogenesis in T cells will be helpful in future developments of diagnostic biomarkers and effective treatments for SLE.

**Figure 1:**
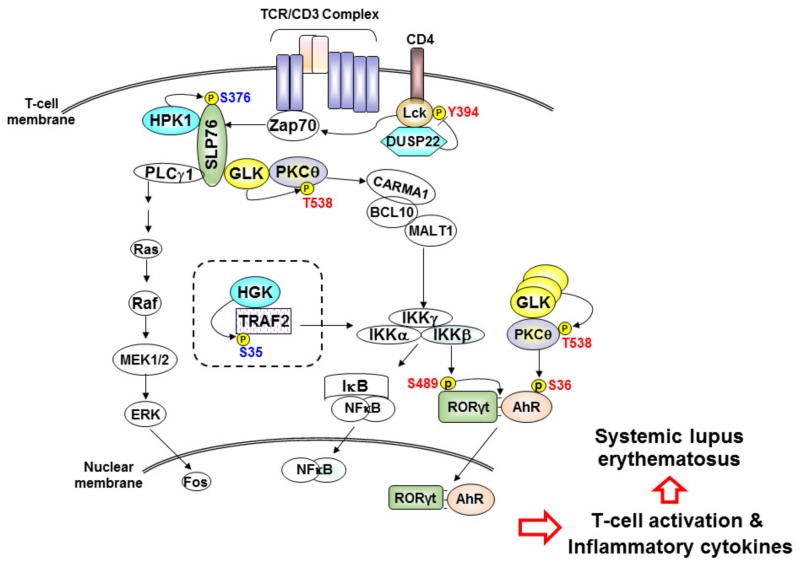
MAP4K1, MAP4K3, MAP4K4, and DUSP22 in T-cell signaling and systemic lupus erythematosus (SLE). The roles of MAP4K1 (HPK1), MAP4K3 (GLK), and DUSP22 (JKAP) in T-cell receptor (TCR) signaling and SLE pathogenesis have been validated using both gene-knockout mice and clinical samples. HPK1 phosphorylates SLP-76 at the serine 376 (S376) residue upon TCR stimulation, resulting in ubiquitin-mediated degradation of SLP-76. HPK1 downregulation in the T cells of human SLE patients leads to the enhancement of T-cell-mediated autoimmune responses. Moreover, DUSP22 (JKAP) dephosphorylates the tyrosine kinase Lck at the tyrosine 394 (Y394) residue, leading to inactivation of Lck and inhibition of T-cell activation. JKAP knockout or deficiency induces T-cell hyperactivation. Consistently, JKAP downregulation in T cells is highly correlated with SLE nephritis and thus is a prognostic biomarker for poor outcome. Furthermore, GCK-Like Kinase (GLK) phosphorylates PKCθ at the threonine 538 (T538) residue, resulting in the activation of the IKK kinase complex and NF-κB. GLK overexpression in T cells further induces interleukin 17A (IL-17A) transcription through the RORγt–AhR complex. IKKβ-induced RORγt serine 489 (S489) phosphorylation and PKCθ-induced AhR serine 36 (S36) phosphorylation result in IL-17A overproduction, leading to autoimmune responses. The GLK-induced SLE pathogenesis has been verified using T-cell-specific GLK transgenic mice and human SLE T cells. In addition, HGK phosphorylates TRAF2 at the serine 35 (S35) residue, resulting in lysosomal degradation of TRAF2. DNA hypermethylation on the HGK promoter results in HGK downregulation and TRAF2 overexpression in T cells of human non-obese type II diabetes patients. DNA methylation of HGK is also changed in human SLE peripheral blood mononuclear cells (PBMCs). HGK levels might also be downregulated in SLE T cells, contributing to autoimmunity. Red residue denotes activating phosphorylation site; blue residue denotes inhibitory phosphorylation site. Arrows denote activation; T bar denotes inhibition. Dashed rectangle denotes potential molecular mechanism for SLE pathogenesis.

Signaling molecules (e.g., kinases and phosphatases) of immune cells play important roles in immune responses and autoimmune pathogenesis through induction of cytokines or chemokines [20,21,22,23,24]. Thus, signaling molecules in T cells are either potential biomarkers or therapeutic targets in the treatment of autoimmune diseases. For example, mitogen-activated protein kinases (MAPKs) are involved in the pathogenesis of autoimmune diseases, including SLE [25]; MAPK inhibitors have been developed for the attenuation of autoimmune responses [20,26]. To date, none of the MAPK inhibitors have progressed to phase III trials due to either lack of efficacy or adverse side effects [27,28]. Studies of these MAPK kinase inhibitors suggest that upstream signaling molecules may be more effective therapeutic targets than downstream signaling molecules [28,29,30]. Similarly, several upstream signaling molecules of MAPK are likely to be potential biomarkers or therapeutic targets for SLE. MAP kinase kinase kinase kinases (MAP4Ks) induce the MAPK c-Jun N-terminal kinase (JNK) through MAP3Ks and MAP2Ks [31,32]. Besides MAP4Ks, MAPK activities are also regulated by dual-specificity phosphatase (DUSP) family phosphatases, which comprise 25 members, including 9 MAPK phosphatases (MKPs) [33,34]. Several MAP4Ks and DUSPs are involved in the regulation of T-cell activation and human SLE. In this review, we summarize the potential utilization of MAP4Ks and DUSPs in T cells as biomarkers and/or therapeutic targets for SLE (Figure 1).

## 2. MAP4K Family Kinases Are Involved in T-Cell Activation and Human SLE

**Figure 2:**
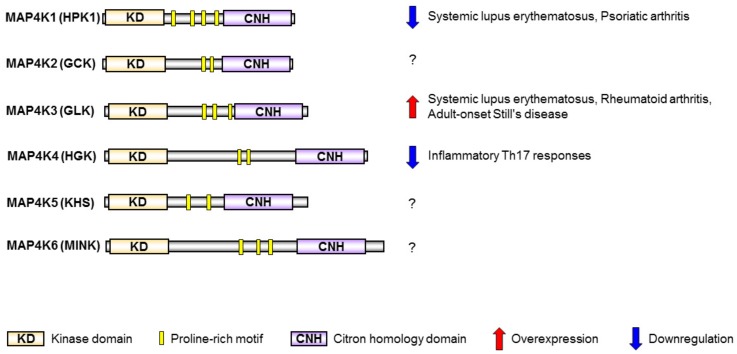
The structural domains and autoimmune-disease involvement of MAP4K family kinases.

MAP4K family kinases—including MAP4K1/HPK1 [35,36,37], MAP4K2/GCK [38], MAP4K3/GLK [39], MAP4K4/HGK [40,41], MAP4K5/KHS [42], and MAP4K6/MINK [43]—are homologous to the mammalian STE20 family of serine/threonine protein kinases. MAP4K family kinases show a high-similarity protein structure, containing an N-terminal kinase domain, several proline-rich regions, and a C-terminal citron-homology domain [31] (Figure 2). MAP4K family kinases are initially identified as upstream molecules that activate MAP3Ks and MAP2Ks, leading to activation of the MAPK JNK [31,32,44,45]. MAP4Ks play important roles in the regulation of cell apoptosis, cell survival, cell autophagy, and cell migration [31,41,46]. Interestingly, several studies reported that MAP4Ks are involved in the regulation of immune-cell responses through JNK-independent pathways [21,22,47,48]. MAP4K1/HPK1 and MAP4K4/HGK play negative roles in T-cell activation and inflammatory responses [21,47]. In contrast, MAP4K3/GLK plays a positive role in T-cell activation and autoimmune responses [10,22]. Moreover, MAP4K1 downregulation and MAP4K3 overexpression in T cells are involved in human autoimmune diseases such as psoriatic arthritis, rheumatoid arthritis (RA), adult-onset Still’s disease, and SLE [22,49,50,51,52] (Figure 2).

### 2.1. HPK1 Transcription Is Reduced in CD4^+^ T Cells of Human SLE Patients

HPK1 (also known as MAP4K1) is a negative regulator of T-cell receptor signaling [21,36]. The HPK1 proteins are cleaved and activated by caspase 3 during apoptosis [53]. HPK1 is also activated by multiple adaptor proteins in mammalian cells, including T cells [36,54,55,56,57,58]. HPK1 directly interacts with and phosphorylates the adaptor protein SLP-76, leading to the inhibition of T-cell activation [21,59] (Figure 1). Notably, HPK1 also phosphorylates the adaptor protein BLNK, leading to the suppression of B-cell activation [48]. Overexpression of HPK1 inhibits T-cell proliferation, T-cell-secreted IFN-γ production, and T-cell-mediated antibody production [49]. Conversely, T-cell receptor (TCR)-induced T-cell proliferation, T-cell-secreted IFN-γ, and T-cell-mediated immune responses are significantly enhanced by HPK1 knockout [21]. Moreover, HPK1-knockout mice display enhanced autoimmune responses and increased CD4^+^ cell infiltration in the central nervous system during the induction of experimental autoimmune encephalomyelitis (EAE) [21]. Consistently, HPK1 mRNA and protein levels are decreased in the CD4^+^ T cells of SLE patients, compared to those of healthy controls [49] (Figure 2). Decreased binding of the Jumonji domain-containing protein 3 (JMJD3) to the HPK1 promoter results in increased H3K27me3 enrichment at the HPK1 promoter in SLE CD4^+^ T cells, leading to inhibition of HPK1 transcription [49]. Similarly, HPK1 is also downregulated in peripheral blood leukocytes in patients with another autoimmune disease—psoriatic arthritis [50] (Figure 2). Th17 cells are involved in the pathogenesis of SLE and psoriatic arthritis. It would be interesting to study the HPK1 inhibition of Th17 differentiation.

Besides HPK1, MAP4K4 (also known as HGK) is a negative regulator of Th17 differentiation [47]. HGK conditional T-cell deficiency results in the induction of inflammatory IL-6^+^ Th17 cells, leading to insulin resistance and systemic inflammation [47] (Figure 1 and Figure 2). Enhancement of HGK DNA methylation and subsequent downregulation of HGK in T cells are biomarkers of Asia-prevalent non-obese type 2 diabetes [60,61] (Figure 1). In addition, DNA methylation profiles of untreated SLE patients indicate that HGK methylation change is associated with SLE manifestations [62]. It is possible that HGK levels are also downregulated in the T cells of SLE patients, contributing to Th17-mediated inflammation.

### 2.2. GLK Is a Biomarker and Therapeutic Target for Human SLE

MAP4K3 (also known as GLK) is an activator of TCR signaling [11,22]. MAP4K3 directly interacts with and phosphorylates PKCθ upon TCR stimulation, resulting in IKK and NF-κB activation [22] (Figure 1). Like HPK1, GLK also interacts with the T-cell adaptor SLP-76, but GLK does not phosphorylate SLP-76. Moreover, SLP-76 is the upstream regulator for GLK kinase activity during TCR signaling [22]. In vitro T-cell proliferation, Th1 differentiation, Th2 differentiation, and Th17 differentiation are impaired by GLK deficiency [22]. GLK-deficient mice display decreased production of T-cell-mediated antigen-specific antibodies and cytokines [22]. Moreover, GLK-deficient mice are resistant to autoimmune disease induction in the experimental autoimmune encephalomyelitis (EAE) mouse model [22]. Consistently, GLK is overexpressed in the peripheral blood leukocytes (PBLs) of SLE patients; the activation of PKCθ and IKK are concomitantly induced in SLE PBLs compared to those of healthy controls [22] (Figure 2). The frequencies of GLK-overexpressing T cells, but not B cells, are increased in SLE patients, compared to those of healthy controls [9,22]. The GLK-overexpressing T cell population is correlated with the SLE disease activity index (SLEDAI) [9,22]. Besides SLE patients, GLK mRNA levels in T cells and GLK-overexpressing T cells are increased in patients with rheumatoid arthritis (RA) and adult-onset Still’s disease, compared to those of healthy controls [51,52] (Figure 2). Moreover, GLK overexpression also occurs in patients with other autoimmune diseases, such as Graves’ disease, Sjogren’s syndrome, and neuromyelitis optica, as well as in patients with cancer recurrence/metastasis [63,64,65,66].

The regulatory mechanisms of GLK overexpression in the T cells of SLE (or other autoimmune diseases) remain unknown. Three microRNAs (let-7c, miR-199-a-5p, and miR-206) have been reported to target GLK 3′UTR in cancer cells [11]; however, it is unclear whether these three microRNAs are decreased in SLE T cells. Enhancement of the long noncoding RNA NEAT induces the production of IL-6, CXCL10, and CCL8 through MAPKs in the monocytes of SLE patients [67]. It is possible that GLK overexpression in SLE T cells is also regulated by long noncoding RNAs. In addition, gene variants of the GLK gene may result in the induction of GLK mRNA levels in SLE T cells. Studying regulatory mechanisms of GLK overexpression in the T cells of SLE (or other autoimmune diseases) may help in the identification of additional therapeutic targets for SLE.

The pathogenic mechanism of GLK-induced autoimmune diseases has been revealed by the data derived from T-cell-specific GLK transgenic mice, plus several knockout mice for individual signaling molecules [10]. GLK overexpression in murine T cells specifically induces production of the inflammatory cytokine IL-17A through the AhR–RORγt complex (Figure 1). GLK signaling induces AhR nuclear translocation and AhR–RORγt complex formation through PKCθ and IKKβ, respectively [10]. In human SLE patients, the GLK^+^IL-17A^+^ CD4^+^ T cell population is drastically increased and is correlated with the SLEDAI [9]. The GLK^+^ Th17 cell population is also a biomarker for identifying active SLE [9]. T cells of SLE and RA patients display induction of GLK-induced AhR–RORγt complex, but healthy controls’ T cells do not [9]. Conversely, treatment of the GLK inhibitor verteporfin efficiently suppresses IL-17A production and AhR–RORγt complex in SLE T cells [9]. Verteporfin treatment also attenuates autoimmune responses in three autoimmune mouse models, including EAE, collagen-induced arthritis (CIA), and T-cell-specific GLK transgenic mice [9]. Collectively, GLK is a biomarker and therapeutic target for autoimmune diseases such as SLE.

## 3. DUSP Family Phosphatases Are Involved in T-Cell Activation and Human SLE

**Figure 3:**
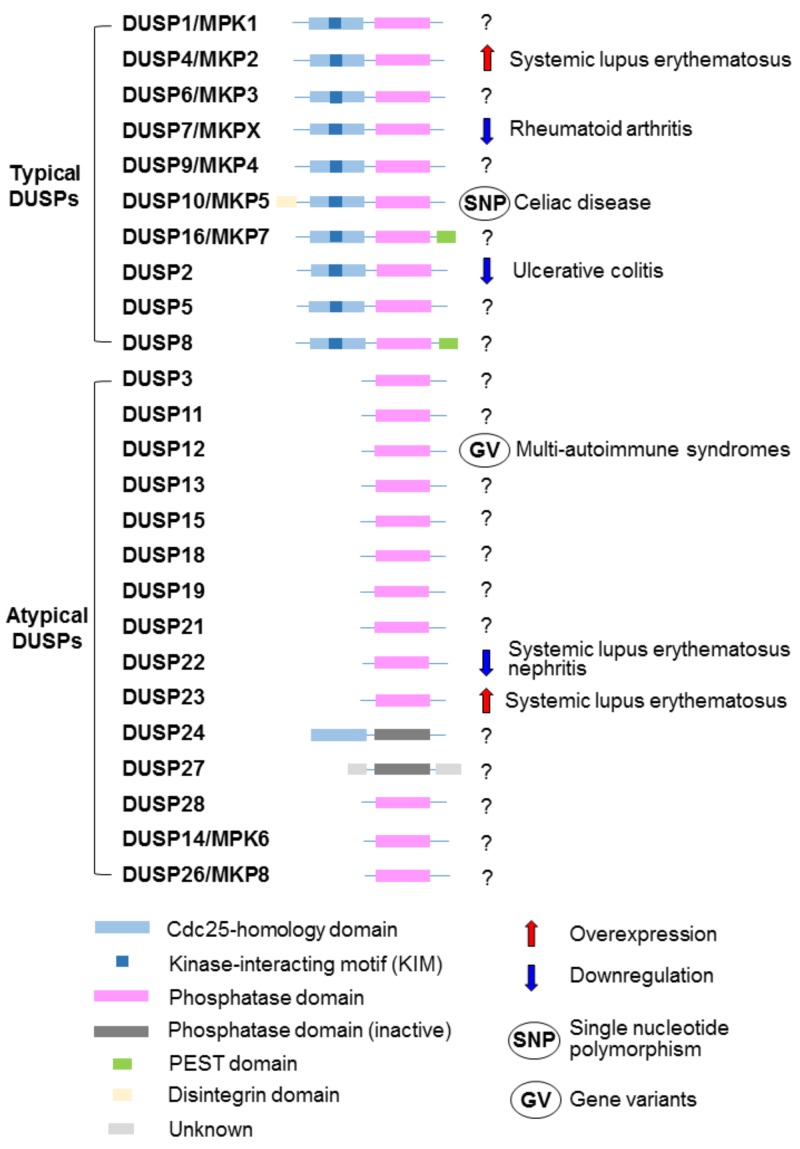
The structural domains and autoimmune-disease involvement of DUSP family phosphatases.

The DUSP family contains 25 phosphatases, which dephosphorylate DUSPs’ substrates at threonine/serine residues and/or tyrosine residues [33,34]. All members of the DUSP family contain a common phosphatase domain [68] (Figure 3). Ten of 25 DUSPs contain the kinase-interacting motif (KIM) or the MAP-kinase-binding motif that interacts with MAPKs [34,69] (Figure 3). These 10 DUSPs are classified as typical DUSPs; 7 of 10 typical DUSPs are named as MAP kinase phosphatases (MKPs) [34] (Figure 3). Another 15 DUSPs do not have KIM and are classified as atypical DUSPs; however, 2 members (DUSP14 and DUSP26) of atypical DUSPs still dephosphorylate MAPKs [34]. Thus, 12 DUSPs have been reported to be MAPK phosphatases. One atypical DUSP, DUSP22, induces the MAPK JNK activation in a phosphatase activity-dependent manner [70]. DUSPs regulate various cellular functions, including cell survival, cell death, cell proliferation, and cell migration [34,71,72]. Several studies reported that DUSPs also regulate immune-cell responses [23,73,74,75,76]. DUSP2, DUSP4, DUSP7, DUSP10, DUSP12, DUSP22, and DUSP23 are involved in human autoimmune diseases, including SLE [77,78,79,80,81] (Figure 3).

### 3.1. DUSP22 Protein Level Is a Diagnostic and Prognostic Biomarker for SLE Nephritis

DUSP22 (also known as JKAP) is an atypical DUSP that activates the MAPK JNK [70]. Besides targeting JNK, JKAP dephosphorylates and inactivates focal adhesion kinase (FAK), leading to the inhibition of cell motility [82]. JKAP also inhibits prostate cancer cell proliferation by reducing EGFR- and androgen-receptor-dependent signaling [71]. Moreover, JKAP plays an inhibitory role in the turn-off stage of TCR signaling by dephosphorylating and inactivating the tyrosine kinase Lck [23] (Figure 1). JKAP-knockout mice display enhanced T-cell-secreted IFN-γ and IL-17A; JKAP-knockout mice are more susceptible to the autoimmune disease induction in the EAE model [23]. Aged DUSP22-knockout mice spontaneously display increased serum levels of pro-inflammatory cytokines (TNF-α, IFN-γ, IL-6, and IL-17A) and autoantibodies (antinuclear antibody and anti-dsDNA) [23]. Consistently, JKAP protein but not mRNA levels are decreased in the peripheral blood T cells of human SLE patients, compared to those of healthy controls [77]. JKAP downregulation in T cells is inversely correlated with daily urinary protein levels of SLE nephritis patients [77] (Figure 3). Moreover, the diagnostic power of JKAP downregulation for active lupus nephritis is higher than that of complements (C3 and C4) and anti-dsDNA antibody levels [77]. A longitudinal observational study further indicates that JKAP downregulation in T cells is correlated with the poor renal outcome of lupus nephritis patients [77]. These findings suggest that JKAP downregulation in T cells is a diagnostic and prognostic biomarker for SLE nephritis. The pathogenic role of DUSP22-deficient T cells in SLE nephritis has been demonstrated by characterizing T-cell-specific DUSP22 dominant-negative transgenic (Lck-DUSP22-C88S Tg) mice [77]. Lck-DUSP22-C88S Tg mice display inflammatory symptoms, including nephritis. Restoration of JKAP expression blocks the induction of IL-17A expression in the T cells of SLE patients [77]. These findings suggest that enhancing either JKAP protein levels or phosphatase activity may help the treatment and attenuation of SLE nephritis.

Protein levels of the tyrosine kinase Lck are decreased in the peripheral blood lymphocytes of SLE patients; however, phosphorylation and activation of Lck are still increased in the T cells of active SLE patients [83,84]. The enhancement of Lck activation is likely due to the JKAP downregulation in active SLE patients.

Besides DUSP22 downregulation in SLE patients, other DUSPs (DUSP2, DUSP7, DUSP10, and DUSP12) are also downregulated or mutated in human autoimmune diseases (Figure 3). The mRNA levels of the Th17 modulator DUSP2 are decreased in the PBMCs of ulcerative colitis patients [75]. DUSP7 mRNA levels are decreased in RA patients [80]. Single-nucleotide polymorphisms on the DUSP10 loci are associated with human celiac disease [85]. DUSP12 gene variants have been identified in patients with multi-autoimmune syndromes, such as the coincidence of Sjögren’s syndrome, RA, and either psoriasis or autoimmune thyroid disease [78]. It would be interesting to study whether DUSP2, DUSP7, DUSP10, and DUSP12 are also involved in human SLE.

### 3.2. DUSP4 mRNA Level Is Increased in CD4^+^ T Cells of Human Juvenile-Onset SLE

DUSP4 (also known as MKP2) is a typical DUSP that inactivates JNK, p38, and ERK [86,87]. DUSP4 overexpression also inhibits STAT5 phosphorylation, whereas DUSP4 deficiency results in enhanced STAT5 phosphorylation/activation in T cells [73]. Moreover, DUSP4-deficient mice show enhanced population of CD4^+^CD25^+^ T (Treg) cells [73]. DUSP4-deficient mice display decreased T-cell-secreted IL-17A; DUSP4-deficient mice are resistant to autoimmune disease induction in the EAE model [88]. Consistently, DUSP4 mRNA levels in differentiated human Th17 cells from healthy donors are higher than those of naïve T cells [79]. Furthermore, DUSP4 mRNA levels of the CD4^+^ T cells from 14 juvenile-onset SLE patients were significantly higher than those of healthy controls [79] (Figure 3). DUSP4 overexpression was associated with high disease activity in 14 SLE patients [79]. The DUSP4 overexpression in SLE T cells is likely due to the enhancement of CREMα/p300-mediated histone acetylation at the DUSP4 gene locus [79]. The data suggest that DUSP4 may be a potential biomarker for juvenile-onset SLE.

### 3.3. DUSP23 mRNA Levels Are Increased in CD4^+^ T Cells of Human SLE

Several genetic variants on human chromosome 1 (1q21–23) have been found to be associated with SLE; these polymorphic genes express inflammation-associated molecules such as C-reactive protein and FasL [89]. Interestingly, DUSP23 is also a gene with polymorphisms on chromosome 1q23. DUSP23 mRNA levels are increased in the CD4^+^ T cells of SLE patients (Figure 3); however, the DUSP23 mRNA levels are not correlated with any SLE clinical parameters [81]. Nevertheless, DUSP23 mRNA levels are correlated with mRNA levels of DNA-methylation enzyme, including DNMT1, DNMT3A, DNMD3B, MBD2, and MBD4 in the CD4^+^ T cells of SLE patients [81]. To date, the role of DUSP23 in the pathogenesis of human SLE remains unclear. Furthermore, the role of DUSP23 in autoimmune responses needs to be validated using DUSP23 knockout and transgenic mice.

### 3.4. DUSP1, DUSP5, and DUSP14 Also Regulate T Cell-Mediated Autoimmune Responses in Mice

DUSP1 (also known as MKP1)-deficient mice display impaired T-cell-mediated immune responses [90]. DUSP1-deficient mice are also resistant to EAE induction; the infiltrating Th17 and Th1 populations are decreased compared to those of wild-type mice [90]. In addition, the induction of CIA was attenuated by DUSP5 overexpression using an electroporation approach in mice [91]. DUSP5-overexpressing mice displayed reduced pro-inflammatory cytokines (IL-1β, IL-6, and TNF-α) in joint tissues and decreased Th17 cells in draining lymph nodes during CIA induction. The attenuation of CIA symptoms may be due to inactivation of STAT3 and ERK by DUSP5 overexpression in CD4^+^ T cells [91]. To date, it remains unknown whether DUSP1 is overexpressed/activated, and if DUSP5 is downregulated/inactivated in the T cells of SLE patients. Nevertheless, the inhibition of DUSP1 and overexpression of DUSP5 may be potential therapeutic approaches for autoimmune diseases, including SLE.

DUSP14 (also known as MKP6) directly dephosphorylates the adaptor TAB1 and inactivates the kinase complex TAB1/TAK1, leading to inactivation of T-cell activation [92]. DUSP14-knockout mice display enhanced T-cell-mediated immune responses; DUSP14-knockout mice are more susceptible to the EAE model than wild-type mice [92]. The phosphatase activity of DUSP14 is induced by the arginine methyltransferase PRMT5-induced methylation and the E3 ubiquitin ligase TRAF2-induced K63-linked ubiquitination of DUSP14 during T-cell receptor signaling [93,94]. Therefore, enhancement/activation of DUSP14 or DUSP14 upstream molecules may lead to the treatment or attenuation of autoimmune diseases.

## 4. Conclusions

Signaling molecules in T cells are dysregulated in SLE patients; downregulation or overexpression of these signaling molecules may be useful diagnostic biomarkers for SLE. Among MAP4Ks and DUPSs, the roles of MAP4K1 (HPK1), MAP4K3 (GLK), and DUSP22 (JKAP) in SLE pathogenesis have been validated using both clinical samples and gene-knockout mice (Figure 1). HPK1 downregulation and knockout result in T-cell hyperactivation enhanced autoimmune phenotypes. JKAP downregulation in T cells is a non-invasive diagnostic biomarker for SLE nephritis and is also a prognostic biomarker for poor outcome in SLE nephritis. Moreover, GLK^+^ Th17 population is a biomarker for active SLE. This GLK^+^ Th17 population will help in the selection of SLE patients that are responsive to GLK inhibitors (e.g., verteporfin), which block RORγt–AhR-complex-induced IL-17A production. Besides inhibition of GLK, activation or overexpression of T-cell signaling suppressors such as DUSP14 and DUSP5 may attenuate inflammatory and autoimmune responses of SLE patients. In addition, the overexpression of DUSP4 and DUSP23 in human SLE T cells as well as the reduction of inflammation in DUSP1-deficient mice suggest that inhibition of DUSP4, DUSP23, or DUSP1 may provide therapeutic benefits for SLE patients. Monitoring the knockout mice for the above potential therapeutic targets of SLE may help to identify any adverse effects caused by inhibiting these targets. A better understanding of additional signaling molecules that regulate T-cell signaling may lead to the identification of novel therapeutic targets for SLE.

## Abbreviations

MAP4K	MAP Kinase Kinase Kinase Kinase
DUSP	Dual-Specificity Phosphatase
HPK1	Hematopoietic Progenitor Kinase 1
GCK	Germinal Center Kinase
GLK	GCK-Like Kinase
HGK	HPK1/GCK-Like Kinase
KHS	Kinase Homologous to Sps1/Ste20
MINK	Misshapen/Nck-Related Kinase
TCR	T-Cell Receptor
PKCθ	Protein Kinase C-theta
IKK	IκB Kinase
JMJD3	Jumonji Domain-Containing Protein 3
H3K27me3	Histone H3 Lysine 27 Trimethylation
PRMT5	Protein Arginine Methyltransferase 5
CREMα	Camp Response Element Modulator α
CIA	Collagen-Induced Arthritis
EAE	Experimental Autoimmune Encephalomyelitis
SLE	Systemic Lupus Erythematosus
RA	Rheumatoid Arthritis
AOSD	Adult-Onset Still’s Disease
SLEDAI	SLE Disease Activity Index
SNP	Single-Nucleotide Polymorphism
